# Successful recovery from coronavirus disease 2019 in a living kidney transplant recipient using low‐dose methylprednisolone

**DOI:** 10.1002/iju5.12226

**Published:** 2020-10-05

**Authors:** Ryo Tanaka, Yoichi Kakuta, Koichi Tsutahara, Masahiro Nakagawa, Naotsugu Ichimaru, Katsuhiko Sakaguchi, Taigo Kato, Ryoichi Imamura, Norio Nonomura, Tetsuya Takao

**Affiliations:** ^1^ Department of Urology Osaka General Medical Cancer Osaka City Osaka Japan; ^2^ Kidney Center Sumitomo Hospital Osaka City Osaka Japan; ^3^ Department of Urology Osaka University Graduate School of Medicine Suita Osaka Japan

**Keywords:** COVID‐19, immunosuppressive therapy, kidney transplantation, low‐dose methylprednisolone, solid organ transplant

## Abstract

**Introduction:**

The data of immunosuppressive therapy management on solid organ transplant recipients with coronavirus disease 2019 are insufficient. We report a kidney transplant recipient who developed coronavirus disease 2019 pneumonia, with successful management of low‐dose mPSL.

**Case presentation:**

A 36‐year‐old man, who underwent living kidney transplantation 1.5 year prior, developed fever. After 10 days, he developed dyspnea, and his blood oxygen levels decreased. Computed tomography showed pulmonary ground‐glass shadow on both lungs, and the coronavirus disease 2019 real‐time polymerase chain reaction test was positive. After reducing the immunosuppressive agents, the C‐reactive protein levels continued elevating, and the pulmonary shadow spread. Subsequently, low‐dose methylprednisolone (40 mg/day) was administered for 4 days and his C‐reactive protein and blood oxygen levels increased and improved, respectively. The coronavirus disease 2019 real‐time polymerase chain reaction test was negative and the pulmonary shadow disappeared.

**Conclusion:**

Low‐dose methylprednisolone may prevent the development of severe coronavirus disease 2019.

**Abbreviations & Acronyms**
COVID‐19: coronavirus disease 2019
CRP: C‐reactive protein
CT: computed tomography
eGFR: estimated glomerular filtration rate
EVL: everolimus
IgA: immunoglobulin A
MMF: mycophenolate mofetil
mPSL: methylprednisolone
O_2_: oxygen
PSL: prednisolone
RT‐PCR: real‐time polymerase chain reaction
SARS‐CoV‐2: severe acute respiratory syndrome coronavirus 2
sCre: serum creatinine
SOT: solid organ transplant
TACER: extended‐release tacrolimus
WBC: white blood cells

**Keynote message**

The strategy of immunosuppressive therapy on SOT recipients with COVID‐19 has not been established. We reported the case of a kidney transplant recipient who developed COVID‐19 pneumonia, with successful management of low‐dose methylprednisolone.

## Introduction

The world is confronting a global novel coronavirus pandemic. Until the end of June 2020, COVID‐19 has been confirmed in >10 million patients, and 450 000 deaths have been reported worldwide. The clinical characteristics, management, and outcomes of COVID‐19 in SOT recipients remain unknown. SOT recipients might have a high risk of complications following infection by viruses, such as the SARS‐CoV‐2, owing to immunosuppression. Herein, we present the case of a kidney recipient who recovered from COVID‐19 following decrease in immunosuppression and administration of low‐dose mPSL. This is the first case of a kidney transplant recipient with COVID‐19 in Japan. It is important to share information regarding COVID‐19 in SOT recipients to establish a treatment strategy.

## Case presentation

**Fig. 1:**
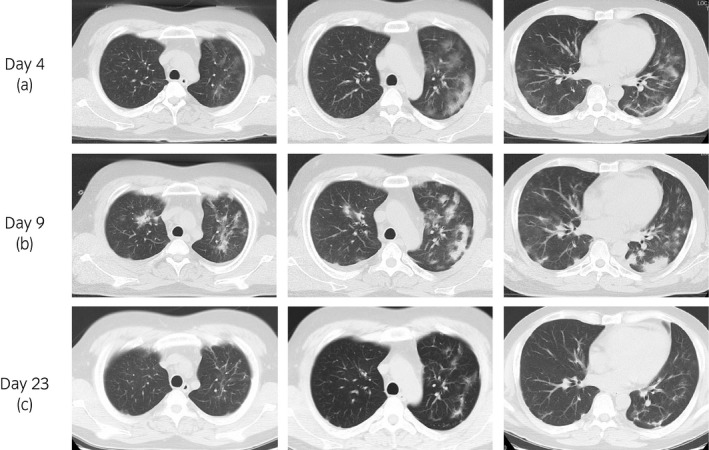
CT images of the patient. (a) On transfer to our hospital (day 4). CT reveals pulmonary ground‐glass shadow on both lungs. (b) Five days after transfer (day 9). CT shows pulmonary shadow getting worse. (c) Six days after low‐dose mPSL administration (day 23). Pulmonary shadow is almost disappeared on CT.

The data of a 36‐year‐old man with end‐stage renal disease owing to IgA nephropathy, who had undergone ABO‐incompatible living kidney transplantation from his father in November 2018, and had sCre levels of 2.0 mg/dL and eGFR of 30 mL/min were examined. His maintenance immunosuppressive regimen was 6 mg of TACER (trough level: 1.6 ng/mL), 1000 mg of MMF, 3 mg of EVL, and 5 mg of PSL. Previous medical history included hypertension and dyslipidemia. He had tonsillectomy for recurrence of IgA nephropathy on the kidney graft in March 2020. He had fever (39.0°C) at 3 days after the surgery. On initial evaluation, the patient did not present with significant signs, except for fever. The blood test showed normal WBC count of 4900 cells/μL, elevated CRP of 9.50 mg/dL, and graft dysfunction (sCre of 3.05 mg/dL). Postoperative fever was noted, and he was followed up with antibiotics. After 10 days, he developed dyspnea, and the blood O_2_ level decreased under 90% in room air. We considered the possibility of COVID‐19 infection. Chest CT showed pulmonary ground‐glass shadow on both lungs (Fig. 1a), and COVID‐19 RT‐PCR test was positive (day 1). The patient was transferred to our hospital for treatment (day 4).

**Fig. 2:**
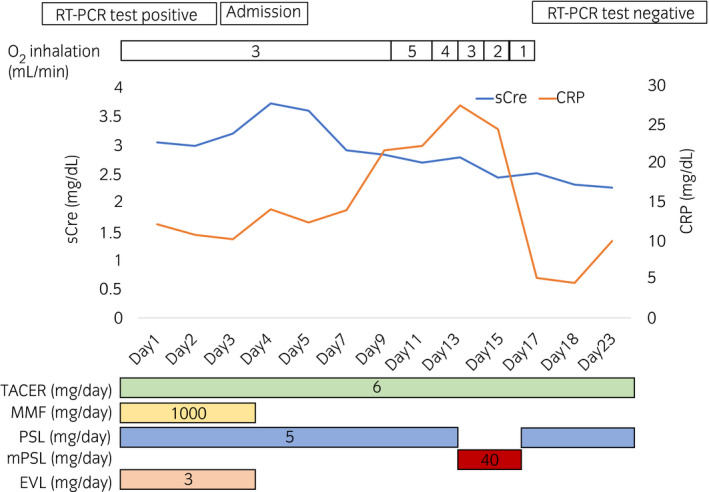
Clinical course of the patient.

Blood test showed normal WBC count of 4700 cells/μL, elevated CRP levels of 14.08 mg/dL, and sCre levels of 3.72 mg/dL. MMF and EVL administration was discontinued, while TACER and PSL administration was continued. He still had fever, CRP continued elevating (Fig. 2), and CT showed that the pulmonary shadow spread (Fig. 1b). Then, low‐dose mPSL (40 mg/day: 0.05 mg/kg/day) was administered intravenously for 4 days (days 14–17), following which, his fever and CRP levels decreased. Moreover, he had improved blood O_2_ levels (98% in room air). COVID‐19 RT‐PCR results were negative and pulmonary shadow was almost disappeared (Fig. 1c). After MMF 1000 mg and EVL 3 mg were resumed on day 32, COVID‐19 RT‐PCR results remained negative (days 24, 28, and 35). His graft function was stable, and the sCre levels were 1.96 mg/dL on day 43.

## Discussion

In the absence of effective antiviral drugs, there are no established treatments for patients with COVID‐19. Corticosteroid treatment has been used in addition to other therapies for patients with severe acute or Middle East respiratory syndrome. However, corticosteroid treatment should not be used for the treatment of COVID‐19‐induced lung injury or shock because they are more likely to cause harm, and they offer no particular benefits.^1^ The current interim guidelines from the World Health Organization unrecommend the use of corticosteroids for the clinical management of severe acute respiratory infection due to COVID‐19, unless indicated for other conditions, such as asthma, chronic obstructive pulmonary disease, or septic shock.^2^ Conversely, the most critically ill patients reportedly tended to use corticosteroids in the clinical settings, and the selection bias and confounders might contribute to increased mortality in those treated with corticosteroids in observational studies.^3^ Moreover, among patients with acute respiratory distress syndrome of COVID‐19, administration of mPSL significantly decreased the risk of death.^4^

The effect of steroid administration may vary depending on the timing of their usage. In the early phase of infection, the symptoms are often revealed relatively mild, and most patients with adaptive immunity cure the disease without exacerbation of infection. Immunosuppressive agents might promote viral replication; therefore, the administration of immunosuppressive agents should be decreased in a SOT recipient with COVID‐19 in early phase. Conversely, in hyperinflammation phase, hyperinflammatory state or cytokine‐release syndrome may cause severe lung injury. Then, several immunomodulation therapies, such as glucocorticoid or anticytokine therapy, are administered, but in this phase, hyperinflammatory state and lung injury may be irreversible. Therefore, steroid administration may benefit patients in the phase before migration to the hyperinflammation phase. Additionally, the corticosteroid dose may be a significant factor of its benefit. In patients with COVID‐19, low‐dose corticosteroid therapy reportedly does not delay viral clearance.^5^ Additionally, patients with severe COVID‐19 pneumonia with early low‐dose steroid therapy (mPSL of 1–2 mg/kg/day) for a short duration (5–7 days) reportedly reduced the O_2_ requirement period and improved the course of the disease.^6^ In this kidney recipient, we discontinued MMF and EVL, but continued TACER and PSL. Then, the CRP levels elevated and the lung injury worsened. Therefore, we administered low‐dose steroid. Subsequently, COVID‐19 pneumonia improved without transition to the hyperinflammation phase and no adverse events occurred. In retrospect, we were stalling steroid administration until day 14 because his O_2_ demand was not high, and his general condition was calm. However, the patient’s CRP levels continuously increased from day 4 and his pulmonary shadow worsened on day 9. It seems that the steroid therapy could have been provided earlier. Additionally, we did not check the T cell counts (CD3, CD4, and CD8) and any inflammation marker, such as interleukin‐6. They may help us infer the state of immunosuppression. TACER was continued to avoid rejection. Additionally, several viruses use active immunophilin pathways during their life cycle, and calcineurin inhibitor may inhibit viral replication *in vitro*.^7^, ^8^ Herein, the authors can make the hypothesis that immunosuppression prevents development of severe COVID‐19.

## Conclusion

We reported the case of a kidney transplant recipient who developed COVID‐19 pneumonia with successful management of low‐dose mPSL. More research is required to determine the usage of immunosuppressive agents in SOT recipients with COVID‐19.

## Conflict of interest

The authors declare no conflict of interest.
